# Provident Dispensaries and the Treatment of Diseases in School Children

**Published:** 1909-01-30

**Authors:** 


### PUBLIC HEALTH AND HYGIENE.

### PROVIDENT DISPENSARIES AND THE TREATMENT OF DISEASES IN SCHOOL CHILDREN.

Provident dispensaries have made but slow progress compared with what might have been expected of them when the claims of the principle they embody are considered. It is obvious from general considerations that the poorer sections of the working classes cannot pay for medical attendance by any system of fees. The smallest reward which the professional man might accept for his services is beyond the resources of those whose earnings barely provide the necessaries of life. When sickness comes their resources are further strained, and with all the will in the world it is frequently impossible to pay the doctor.

Some system of insurance is indicated as inevitable if the medical man is not to be victimised, as for so long he has been. Hospital abuse in the fullest sense of the term is only one of the evils which have sprung from the lack of organisation of other means of provision of medical attendance. One of the chief advantages of the medical inspection of school children has been to quicken, in anticipation of its results, the public sense of the need for the better organisation of medical service. The power to provide for such attendance which is now vested, subject to the approval of the Board of Education, in the local authorities, and for which the rates stand forfeit in the absence of other sources of revenue, has deepened the public interest in matters affecting the medical attendance of the poor. It is already seen that when the full extent of neglected disease is laid bare the question of better provision than has satisfied the public conscience in the past will inevitably have to be faced. We are glad to note that in many districts the loom of this question has set going timely attempts to improve the admittedly inadequate existing methods.

The extension of the provident dispensary system offers the prospect of a satisfactory solution of the problem. The provident dispensary in essence is a mutual insurance against sickness unprovided with medical care. It has been found in practice to work satisfactorily, medical men and patients alike being content with its results. But harmony has only been secured by the definite observance of certain rudimentary principles which it is most desirable should receive recognition wherever the extension of the dispensary system is contemplated. The first of these considerations is that the dispensary patient should be free to select his own medical attendant, and that his choice should include all the practitioners established in the area provided for by the dispensary. A corollary to this recognition of the patient's freedom is that the practitioner should retain the right to refuse to admit to his list any applicant to whom he may take exception. This mutual freedom of patient and practitioner carries into contract practice that liberty of action which with all its drawbacks is yet one of the chief merits of private practice. It goes without saying that in many of its minor details the working of this principle requires regulation, but it is an ingrained prejudice of the average Englishman to distrust officials other than those of his own choosing, and it is doubtful whether there is any feature in the practitioner's life which he more values than his independence of all but self-imposed restraints. Both parties to the contract are therefore satisfied when such liberty of choice and action are secured.

The next most important consideration relates to the financial basis of the dispensary. There are two safeguards against abuse of the provident dispensary; one is to insist upon a wage limit for all beneficiaries; the other to adopt a sliding scale of payments graduated according to income. The safeguard hitherto chiefly adopted has been to insist upon a wage limit which has usually ranged nominally to a maximum of £2 a week. It is questionable whether this range might not be considerably widened if the contributions of those in receipt of larger incomes were fixed on a more adequate scale. The doctor's bill is more easily paid where it is anticipated by weekly or monthly contributions, systematically collected during health, rather than after sickness has strained the normal resources of the patient. The actuarial question of what would be the equivalent of the average fees paid to doctors by families in the middle and better-to-do working classes does not seem incapable of solution, and if the contributions of persons at present excluded from the benefits of the provident dispensary were determined on this basis the general practitioner would certainly not be worse off as a result of such organised provision. What is clear is that if a rate-aided provision is made under the powers conferred by the Education Administrative Provisions Act, it will be taken advantage of by many persons who at present are excluded from participating in the benefits of the provident dispensary.

Provident dispensaries might be so organised as to make the conditions of medical practice much more satisfactory than they are at present. The contributions should be such that the sum received by the practitioner should amply reward him for the services he is called upon to render in his relation to the institution, and should not be less than he receives in average private practice for identical work. If the better-to-do are admitted on such a basis, no one will have cause to complain. The class in whose interests provident dispensaries are chiefly organised do not contribute so as to secure this result, and the charitable—of whom the medical practitioners are chief—have to make good the balance.

Self-supporting provident dispensaries, in other words, mean dispensaries which, while maintaining themselves by the contributions of the subscribers, do so only at the cost of inadequately rewarding the practitioner. They are much better than no provision at all, for then the practitioner is even less rewarded for his services, but they still throw an undue burden upon the medical practitioner.

The efforts of the workman to provide medical attendance for himself and family should therefore be supplemented by the gifts of the charitable, and if need be by aid from the rates.

The problem of the treatment of disease in school children will probably find its most satisfactory solution in some such compromise as is here foreshadowed. There will be a strong inducement for the working classes to join some organisation in which their contributions leave them with a sense of comparative independence while the supplementary contributions place within their reach a practically unrestrained choice of medical attendants whose services are secured on terms which are at the same time an inducement to the practitioner.

The local education authorities on whom the burden of providing treatment is likely to be thrown in the absence of some such provision as this will probably welcome the provident dispensary as an alleviation of their prospective burden, and the time seems opportune for medical practitioners, the local education authorities, and the general public to take common action in organising in each centre of industry some better provision for the medical care of the working classes.

The new provident dispensary opened in Birmingham on January 1, though falling short in its organisation of all that a provident dispensary might be, includes some of the best features of these institutions. Mr. Neville Chamberlain has been chosen its first president. The co-operation of the medical profession has been invited and secured, and circulars have been issued to employers of labour asking them to cause to be posted in a prominent place in their factories or workshops a bill setting forth advantages of membership, which are stated as follows:—

(1) Free choice of medical attendant from among a number of the doctors in your own neighbourhood. (2) Attendance in your own home if you are not well enough to attend at the surgery. (3) Medicine supplied by the nearest branch of the General Dispensary. (4) Advice, of consulting physicians or surgeons in the city on payment of reduced fees. (5) Special rates of subscription for families of over four members.

The question of government of the institution will be vested in a central committee, which will be constituted of seven members of the provident dispensary, elected annually from among the adult members of at least one year's standing, one member being elected from each district. Seven members of the medical profession, to be nominated in the first instance by the General Practitioners' Union, but subsequently to be elected annually, one from each district, by the members of the provident dispensary staff. Seven representatives appointed by the Birmingham branch of the British Medical Association; four hospital representatives—namely, one each from General, Queen's, Children's and Homœopathic Hospitals; two representatives from the General Dispensary; one from the City of Birmingham Aid Society; one from the Birmingham Hospital Saturday Fund; together with a president and an hon. secretary, to be elected by the committee.

Although Birmingham does not on this occasion lead the way, it has followed an excellent example, and in wishing the adventure the success we believe to be assured, we venture to express the hope that other towns will not be slow in taking timely steps of a like character, which are calculated to benefit mutually both the medical profession and the public.

